# Has the fall of *Roe* changed contraceptive access and use? New research from four US states offers critical insights

**DOI:** 10.1093/haschl/qxae016

**Published:** 2024-02-08

**Authors:** Megan L Kavanaugh, Amy Friedrich-Karnik

**Affiliations:** Research Division, Guttmacher Institute, New York, NY 10038, United States; Research Division, Guttmacher Institute, New York, NY 10038, United States

**Keywords:** *Dobbs v. Jackson Women's Health Organization*, contraception, health policy, health care access, sexual and reproductive health care

## Abstract

The overturning of *Roe v. Wade* in the 2022 *Dobbs v. Jackson Women's Health* *Organization* decision has had vast impacts on abortion access across the United States, but less is known about the wider impacts on people's contraceptive access. We draw on cross-sectional survey data representative of reproductive-aged women in Arizona, Iowa, New Jersey, and Wisconsin at two time points—one prior to and one following the *Dobbs* decision. We examined changes between these two time points in key sexual and reproductive health metrics and, at the post-*Dobbs* time point, differences in these metrics across age, sexual and gender minority status, nativity, and income status. Between these two time points, we found statistically significant evidence that sexual activity declined, barriers to accessing contraception increased, reports of receiving high-quality contraceptive care decreased, and condom use increased. As continued fallouts of the *Dobbs* decision on access to abortion occur, this research makes clear that access to broader contraceptive care is worsening. Policies that promote meaningful access to all forms of sexual and reproductive health care must be advanced to support all individuals' right to reproductive autonomy while mitigating inequity and inequality.

## Teaser

While it is well documented that abortion access in the United States has been upended since the Supreme Court's 2022 *Dobbs v. Jackson Women's Health Organization* decision, we know less about the wider impacts on people's contraceptive access since the decision. This Brief Report starts to shed light on that by drawing on data from surveys of reproductive-aged women in Arizona, Iowa, New Jersey, and Wisconsin prior to and following the *Dobbs* decision to examine changes in key sexual and reproductive health metrics. Between these 2 time points, we found that sexual activity declined, barriers to accessing contraception increased, reports of receiving high-quality contraceptive care decreased, and condom use increased. The data also reveal that people who have always experienced the greatest barriers to sexual and reproductive health care, especially young people, queer people, low-income people, and foreign-born people, continue to have the most challenges in the post-*Dobbs* era. We need to respond with policies that promote meaningful access to all forms of sexual and reproductive health care while mitigating inequity.

## Introduction

A rapidly growing body of research highlights how the overturning of *Roe v. Wade* in June 2022 has led to massive disruptions in sexual and reproductive health (SRH) care, from upending abortion access in entire regions of the country^1^ to worsening an already dire maternal health crisis in many states.^2^ One issue that is less well understood is how the *Dobbs v. Jackson Women's Health Organization* Supreme Court decision may have contributed to changes in people's SRH outcomes beyond abortion. Since decisions around abortion are not isolated from decisions about the rest of one's reproductive health, we anticipate that the *Dobbs* decision could have much broader consequences for people's reproductive autonomy—including potentially shaping attitudes toward becoming pregnant, changing their sexual activity and contraceptive strategies, and impacting their ability to access necessary health care. Although a variety of media reports have highlighted anecdotal stories and small studies describing just these sorts of changes,^3-5^ more population-based evidence is warranted.

## Data and methods

The Reproductive Health Impact Study^6^ is a multistate research initiative covering 2017–2024, which is tracking the impact of state- and federal-level policies on publicly supported family planning networks and the patients who rely on this care. The initiative focuses on four states selected because of anticipated or realized shifts in public funding streams to SRH care and with varying climates of support for SRH during the overall study time frame: Arizona (less supportive, limited to no abortion access post-*Dobbs*), Iowa (less supportive, limited to no abortion access post-*Dobbs*), New Jersey (more supportive, ongoing access to abortion post-*Dobbs*), and Wisconsin (less supportive, limited to no abortion access post-*Dobbs*).^7^ We draw on data collected via randomly sampled, household-based, cross-sectional surveys of women (we use “women” throughout this piece to reflect the terminology and recruitment processes used for this data-collection effort, but we recognize that the *Dobbs* decision has implications for the reproductive autonomy of all people with a capacity for pregnancy, including those identifying across the gender spectrum) ages 18–44 years in the four states^8^ at two time points—one prior to the *Dobbs* decision (data collected between February and July of 2021) and one following the *Dobbs* decision (data collected between September 2022 and August 2023). Sample sizes for each state survey at each time point ranged from about 1200–2200 respondents. We focus on eight key SRH metrics measured at both time points: importance of avoiding pregnancy now, frequency of penile-vaginal sex in the past three months, experience of trouble or delays in obtaining desired contraception in the past 12 months, receipt of contraceptive care in the past 12 months, experience of high-quality contraceptive care from the health care provider seen most recently in the past 12 months,^9^ overall contraceptive use in the past three months, specific method use in the past three months, and current use of preferred contraception. We use simple logistic regression to examine changes from the pre- to post-*Dobbs* time points in key SRH metrics by state and, at the post-*Dobbs* time point, within specific population groups across the four states who have historically experienced greater barriers to SRH care (by age, sexual and gender minority status, nativity, and income status). We consider changes between the two time periods to be significant at the *P* < .05 level. Data are weighted to be representative of the reproductive-aged population of women in each state at each time point.

## Results

### Changes in pregnancy attitudes and sexual activity

We found no evidence of change in pregnancy attitudes among reproductive-aged women in Arizona, Iowa, New Jersey, and Wisconsin during this time frame. We do, however, note declines in sexual activity in Iowa, New Jersey, and Wisconsin that continue downward trends already documented prior to the *Dobbs* decision.^10^ These declines are based on increases of 6 to 8 percentage points across these three states in the proportion of respondents indicating they had no penile-vaginal intercourse in the prior three months, with no corresponding changes in relationship statuses occurring between the two time points.

### Changes in access to contraceptive care

**Figure 1.**
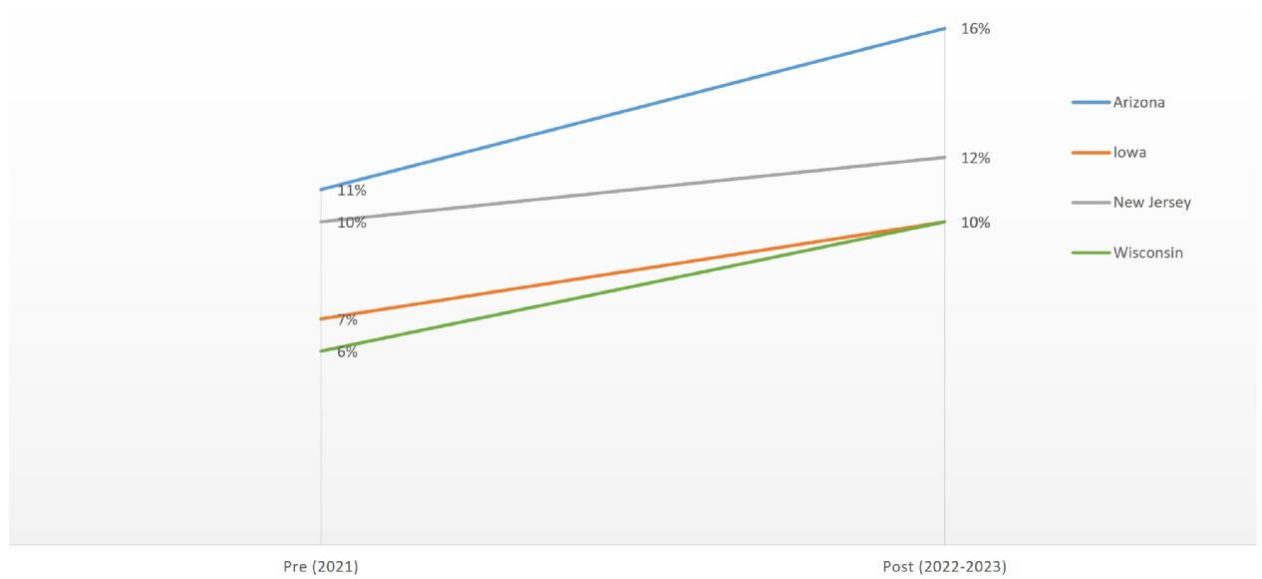
Percentages of reproductive-aged women reporting troubles or delays in accessing their preferred contraception, pre- and post-*Dobbs.* Source: authors' analysis. All post-*Dobbs* data points represent statistically significant differences from the pre-*Dobbs* data points at the *P* < .05 level in Arizona, Iowa, and Wisconsin.

Across these four states, we found significant changes in individuals' reports of access to and experiences with contraceptive care (receiving recent contraceptive care includes discussing information about, and/or having a check-up or medical test related to, and/or receiving a contraceptive method or prescription during the 12 months prior to time of survey). Between the pre- and post-*Dobbs* survey time points, in Arizona, Iowa, and Wisconsin, we found increases of approximately 4 percentage points in the percentage of reproductive-aged women who reported trouble and/or delays in accessing their preferred contraception (Figure 1). There was no significant change in these reported barriers among reproductive-aged women during this same time period in New Jersey.

**Figure 2.**
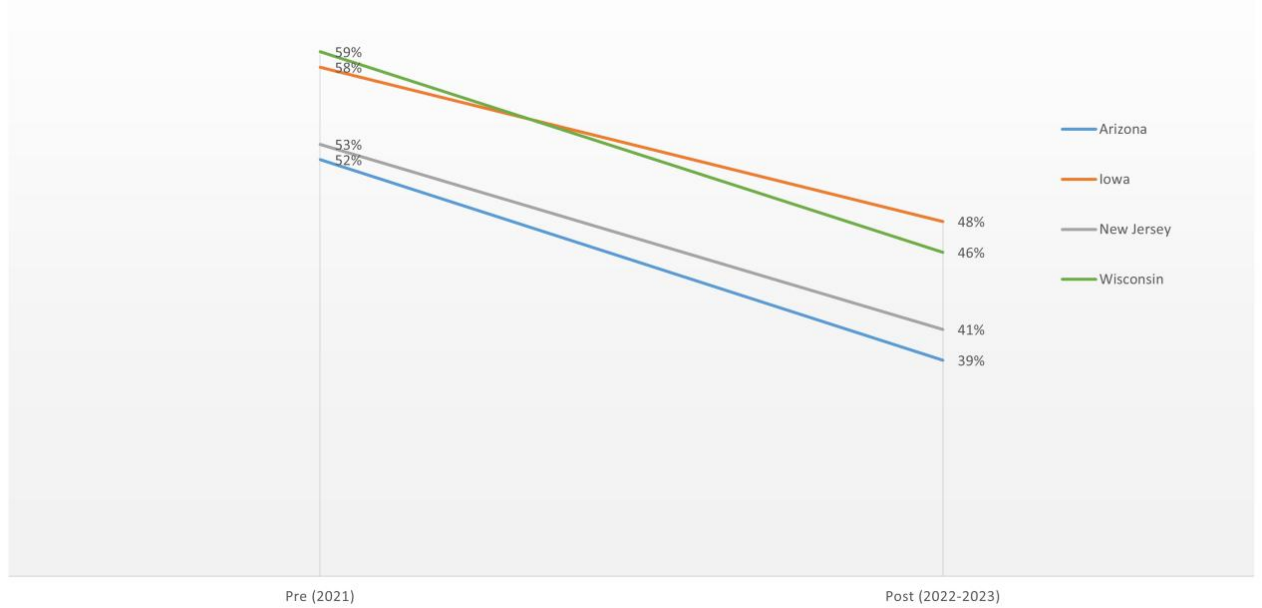
Percentages of reproductive-aged women reporting receiving high-quality contraceptive care, pre- and post-*Dobbs.* Source: authors' analysis. All post-*Dobbs* data points represent statistically significant differences from the pre-*Dobbs* data points at the *P* < .05 level in each of the four states.

We also found between 6- to 12-percentage-point decreases in the percentage of reproductive-aged women in Iowa, New Jersey, and Wisconsin reporting having received recent contraceptive care. Among those who received recent contraceptive care in all four states, fewer reported that the care they received was high quality (Figure 2), indicating that patients experienced this care as being less person-centered (quality of contraceptive care in this analysis was determined based on responses to four survey items that make up the person-centered contraceptive counseling [PCCC] scale).^8^ Person-centered care is respectful of, and responsive to, individual patient preferences, needs, and values, ensuring that patients’ values guide clinical decisions^11^ over time.

### Changes in contraceptive use

When examining the use of specific methods, reproductive-aged women in three of the four states—Iowa, New Jersey, and Wisconsin—reported increases in use of condoms. The research did not reveal significant changes in the proportion of each state's reproductive-aged population using any other methods. We found no significant changes in reports of use of intrauterine devices (IUDs) or permanent methods—either vasectomy or tubal ligation—in contrast to media reports highlighting how requests for these methods and services increased in the post-*Dobbs* landscape.^12,13^ And, as with contraceptive use overall, we did not find evidence that the proportion of women who were using their preferred contraceptive method changed between the two time points in any of the four states.

### Post-*Dobbs* differences in outcomes across population groups

In comparing the experiences of young people ages 18–24 with those of people ages 25–44 in the post-*Dobbs* landscape, we found that young people reported higher levels of trouble accessing preferred contraception (17% vs 11%), higher levels of receiving contraceptive care (49% vs 34%), and lower levels of receiving high quality, person-centered care (34% vs 47%). Young people also reported lower levels of overall contraceptive use (68% vs 74%), higher levels of use of both condoms (55% vs 34%) and emergency contraception (EC) (17% vs 5%), and lower levels of using their preferred contraception (55% vs 61%) than their older counterparts.

Women born outside of the United States reported lower levels of receiving recent contraceptive care (31% vs 39%), less frequent contraceptive method use (72% vs 80%), higher levels of condom (48% vs 38%) and IUD (27% vs 20%) use, and lower levels of using preferred contraception (45% vs 62%) in the post-*Dobbs* time period compared with those born in the United States.

Individuals who identified as a sexual or gender minority reported higher levels of trouble or delays in getting their preferred contraception (18% vs 11%), lower levels of using contraception (66% vs 75%), and lower levels of using their preferred contraception (52% vs 62%) in the post-*Dobbs* period than did those who identified as cisgender and/or straight during the same time period.

Those living at less than 200% of the federal poverty level reported higher levels of trouble and delays getting their preferred contraception (17% vs 11%), lower quality contraceptive care (38% vs 45%), lower levels of using contraception overall (71% vs 75%), less frequent method use (70% vs 83%), higher levels of condom (43% vs 37%) and EC (13% vs 7%) use, and lower levels of preferred method use (51% vs 63%) than those living at higher income levels in the post-*Dobbs* period.

## Discussion

As we continue to experience the fallout of the *Dobbs* decision on access to abortion, this research makes clear that many people are also experiencing worsening contraceptive care. While the changes in SRH outcomes highlighted in this research cannot be directly attributed to the *Dobbs* decision, taken in tandem with a growing body of evidence on people's changing contraceptive strategies and increased barriers to SRH care,^14,15^ they help to paint a picture of a health care system continuing to struggle to meet people's reproductive needs, even in states supportive of reproductive health and rights.

In the four states we studied, people with the capacity to become pregnant experienced decreases in their overall access to contraceptive care and decreases in the quality of that care when they received it. The increases in condom use may be reflecting difficulties that individuals are increasingly having in accessing methods they depend on the health system to provide. Despite anecdotal reports in the media to the contrary, we did not find evidence of large shifts in pregnancy attitudes or specific method use changes other than condoms in these four states between these two time points.

The period we studied overlaps with the aftermath of the COVID-19 pandemic and Trump administration attacks on the US family planning safety net. Our analytic approach does not allow for causal interpretation of these findings so, while not all changes noted can necessarily be directly attributed to the *Dobbs* decision and there may be unobservable differences across states at play, this emerging evidence is nevertheless vital to help shape evidence-based policies and programs to better meet people's contraceptive needs in the post-*Dobbs* era, shore up their access to reproductive health care more broadly, and affirm their right to reproductive autonomy.

Furthermore, the data reveal that people who have always experienced the greatest barriers to realizing their reproductive autonomy, especially young people, queer people, low-income people, and foreign-born people,^16-18^ are bearing the brunt of the multiple, co-occurring disruptions. While individuals have some—and varying—levels of agency regarding their attitudes towards pregnancy, decisions around sexual behavior, and what contraceptive methods to use, these individual-level intentions and behaviors cannot be realized without broad systems-level shifts that center people's needs and desires in their health care.

## Conclusion

Removing barriers to desired health care and ensuring reproductive autonomy for all must be the goals of our SRH care system.^19^ Our health care system, including the policies and incentives that drive the provision of health care, must center patients' needs—especially the needs of those who have historically borne the brunt of restrictive policies—in the provision of care, recognizing that these needs shift and evolve over time. Increasing access to contraception is certainly not the fix-all solution to broken abortion access; indeed, the very act of siloing out one reproductive life event from others is a losing strategy for attending to people's overall SRH and well-being. Solutions lie on the path of treating contraceptive care and abortion and pregnancy and infertility and the full range of SRH as critical to people's full well-being.

At a minimum, policymakers must ensure that all public and private health insurance plans and programs cover the full range of reproductive health care, including over-the-counter options, without cost sharing, as well as cover person-centered counseling on comprehensive SRH, including all contraceptive methods and abortion. Recognizing the need for action, the current administration issued an Executive Order^20^ in June 2023 to broaden access to contraception, signaling that the federal government recognizes the dire situation of our health care infrastructure with regard to SRH. The priorities spelled out in that Order must be fully realized and other policies that promote meaningful access to these types of care must be advanced to support all individuals' right to reproductive autonomy while mitigating inequity and inequality.

Going forward, continuing to track how, and whether, these initial changes in people's SRH behaviors, experiences, and access seen in the early months after the *Dobbs* decision are maintained or whether they become exacerbated as restrictions on abortion access and reproductive freedom more generally grow will be a critical endeavor to informing the best paths forward.

## Supplementary Material

qxae016_Supplementary_Data
